# ISCEV extended protocol for the dark-adapted red flash ERG

**DOI:** 10.1007/s10633-018-9644-z

**Published:** 2018-06-22

**Authors:** Dorothy A. Thompson, Kaoru Fujinami, Ido Perlman, Ruth Hamilton, Anthony G. Robson

**Affiliations:** 1 The Tony Kriss Visual Electrophysiology Unit, Clinical and Academic Department of Ophthalmology, Great Ormond Street Hospital for Children NHS Trust, London, UK; 2 UCL Great Ormond Street Institute for Child Health, London, UK; 3 Laboratory of Visual Physiology, Division for Vision Research, National Institute of Sensory Organs, National Hospital Organization, Tokyo Medical Centre, Tokyo, Japan; 4 Department of Ophthalmology, Keio University School of Medicine, Tokyo, Japan; 5 UCL Institute of Ophthalmology, London, UK; 6 Ruth and Bruce Rappaport Faculty of Medicine, Technion-Israel Institute of Technology, Haifa, Israel; 7 Department of Clinical Physics and Bio-Engineering, Royal Hospital for Children, NHS Greater Glasgow and Clyde, Glasgow, UK; 8 College of Medical, Veterinary and Life Sciences, University of Glasgow, Glasgow, UK; 9 Electrophysiology Department, Moorfields Eye Hospital, London, UK

**Keywords:** Clinical standards, Electroretinogram (ERG), Full-field ERG, International Society of Clinical Electrophysiology of Vision (ISCEV), Dark-adapted (DA), Red flash ERG, Retinal dystrophy

## Abstract

The International Society for Clinical Electrophysiology of Vision (ISCEV) standard for full-field electroretinography (ERG) describes a minimum procedure, but encourages more extensive testing. This ISCEV extended protocol describes an extension to the ERG standard, namely the dark-adapted (DA) red flash ERG. The DA red flash ERG can be incorporated conveniently within the ISCEV standard ERG protocol after a minimum of 20-min DA and recorded after the DA 0.01 ERG to a flash strength of 0.3 phot cd s m^−2^, eliciting a waveform with two positive peaks in healthy individuals. The first positive component is the cone-mediated x-wave with a peak at 30–50 ms; the second is a rod-mediated b-wave with a peak time of approximately 100 ms. Shorter DA times may be desirable to shorten the recording time or to alter the prominence of the early cone-mediated x-wave relative to the rod-mediated b-wave. The DA red flash ERG is used to aid the diagnosis of achromatopsia (rod monochromacy), cone dystrophy and other forms of cone system dysfunction, including “Bradyopsia” (RGS9/R9AP-retinopathy), when the DA red flash ERG x-wave is preserved in the absence of ISCEV standard LA ERGs. The DA red flash ERG can also help determine the origin of residual DA ERGs in cases of severe rod dysfunction, for example in disorders such as vitamin A deficiency, fundus albipunctatus (RDH5-retinopathy), Oguchi disease (SAG- or GRK1-retinopathy) and some rod-cone dystrophies. To shorter DA periods, the x-wave may be elicited without the following rod b-wave, shown to be helpful in abbreviated protocols for children.

## Introduction

The International Society for Clinical Electrophysiology of Vision (ISCEV) standard for full-field electroretinography (ERG) describes a minimum set of tests, but encourages the use of additional ERG protocols for clinical ERG testing [1]. This extended protocol describes the dark-adapted (DA) red ERG, as a specialized procedure which is well established and broadly accepted by experts in the field. The protocol was prepared by the authors in accordance with ISCEV procedures (http://www.iscev.org/standards/index.html) and was approved by the ISCEV Board of Directors on March 25, 2018.

## Scope and applications

The ISCEV ERG standard [1] describes a minimum protocol to test generalized rod and cone system function in the outer and inner retina. The DA red flash ERG can be used to distinguish the function of DA rod and cone systems and can help determine the origins of abnormal standard flash ERGs, which may be important for accurate characterization of retinal function and to establish some diagnoses. This extended protocol describes parameters for the dark-adapted (DA) red flash ERG that may be added to the ISCEV standard ERG protocol.

The normal cone system contributes to the full-field ERG under DA as well as light-adapted (LA) conditions. This occurs in DA ERGs evoked by flash strengths greater than 0.1 cd s m^−2^ [2], including the ISCEV standard DA 3 (“combined rod cone”) and DA 10 (“strong flash”) ERGs. Early investigations revealed the contribution of DA cones in the ERG waveform by using colored flashes that exploited differences in the spectral sensitivities of rods and cones [3–5]. These studies showed that the DA ERG waveform to a red flash has two distinct positive peaks. The first, named the x-wave, occurred within 30–50 ms and was attributed to DA cone activity. The x-wave was followed by a rod-mediated b-wave [3]. The x-wave is larger than the b-wave during the early stages of dark adaptation when the rod system threshold is high. As dark adaptation proceeds, the x- and b-wave amplitudes become similar and finally the b-wave exceeds the x-wave [6].

The DA red flash ERG has several clinical applications, and the circumstances and diagnoses that may benefit from testing are outlined below:

- The DA red flashes are usually well tolerated by patients of all ages, and the test is therefore useful if photophobia or photo-aversion confounds the recording of standard LA ERGs. This can occur in the presence of cone dysfunction, but also, for example, in the presence of media opacity or strong Bell’s phenomenon.
- In cases of generalized cone system dysfunction such as rod- and S-cone monochromacy and cone dystrophy, the DA red flash ERG x-wave may be undetectable, markedly attenuated and/or delayed [7–10].
- In cases of generalized retinal dysfunction, the relative involvement of the DA red flash ERG x-wave and b-wave may suggest predominant dysfunction of cone or rod systems, not always obvious by comparing standard DA and LA ERGs.
- In cases of severe or selective rod dysfunction, the DA red flash ERG can help determine the causes and origins of abnormal or residual DA bright flash ERGs. This occurs, for example, in vitamin A deficiency [11], fundus albipunctatus (RDH5-retinopathy) [12] and Oguchi disease (SAG- or GRK1-retinopathy) [13] and in some cases of rod-cone dystrophy including early stages of Bothnia dystrophy (RLBP1-retinopathy). In these disorders, the DA 3 and DA 10 ERGs have reduced a-waves indicating rod photoreceptor dysfunction, but there may also be reduction in the b:a ratio and shortening of b-wave peak time in the absence of a rod system contribution. The reduced b:a ratio may arise from strong stimulation of the relatively preserved DA cone system, analogous to the photopic hill phenomenon, and produces a b-wave which resembles the DA red flash ERG x-wave.
- “Bradyopsia” (RGS9- and R9AP-retinopathy). The DA red flash ERG is normal, but LA cone-mediated ERGs are extinguished by repetitive flashes [14, 15]. The combination of a preserved DA red flash ERG x-wave and undetectable or severely abnormal standard LA ERGs is pathognomonic for the disorder.
- The red flash ERG has been used to detect color vision deficiencies and has been reported to be absent [9, 16] or subnormal [10] in protanopia. The implication is that around 1/100 males would have an absent red flash ERG although this has not been established for an ISCEV DA red flash ERG extended protocol.

## Patient population

Patients of all ages, referred for investigation of possible retinal dysfunction, retinal dystrophy, generalized cone or rod system dysfunction or patients with photophobia may benefit from the DA red flash ERG.

## Technical issues

The DA red flash ERG will follow the specifications of the current ISCEV standard full-field ERG and for most applications may be embedded within the standard protocol [1].

**Fig. 1:**
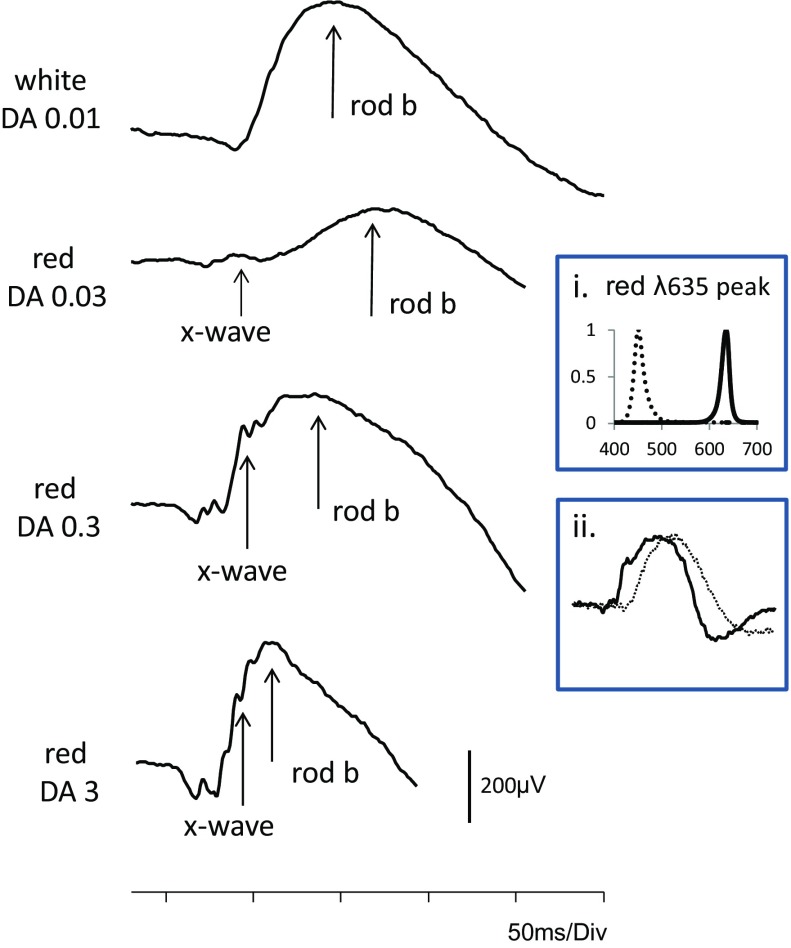
An ISCEV standard DA 0.01 ERG is shown at the top of the figure to compare the waveform of the rod driven b-wave with that of DA red flash ERGs produced by three different flash strengths of wavelength 635 nm after 20-min dark adaptation. Note the separation in peak time of the x-wave and b-wave to DA 0.03 cd s m^−2^ (dim) flashes, the enlargement of the x-wave to DA 0.3 cd s m^−2^ and the merging of x- and b-waves at DA 3 in a control subject. Insert i shows the spectral characteristics of the red (solid line) and blue (dotted line) LEDs in the Ganzfeld. Insert ii shows the DA red flash ERG to 0.3 cd s m^−2^ in a second subject compared with a DA blue flash ERG of “scotopically matched” b-wave amplitude, in this case DA blue 0.0003 cd s m^−2^ (the red flash response may also be “matched” to a DA dim white flash ERG). DA red ERG is shown as a solid black lines and DA blue flash ERG as a dotted line

Additional considerations include the following:

- The spectral characteristics of the red flash. Both peak wavelength and bandwidth may affect the DA red flash ERG. Physical filters, e.g., Kodak Wratten filters 26 (dominant wavelength 619 nm) or 29 (dominant wavelength 630 nm), were used in many older studies, but have been largely superseded by LEDs, e.g. peak wavelengths 635 or 655 nm, and choice may be equipment dependent. It is noted that peak wavelengths shorter than 620 nm may be perceived as orange, and wavelengths longer than 630 nm provide slightly better separation of x-wave and b-wave, and that for wavelengths longer than 650 nm waveforms have been reported with a third positive wave, later than the rod b-wave [6].
- The units of flash strength. The relative (effective) strength of a colored flash depends upon the adaptation and hence spectral sensitivity of the eye. Absolute measures are radiant energy, but for uniformity of clinical use and consistency with other flash stimuli, photometric units defined in phot cd s m^−2^ are recommended.
- Duration of dark adaptation. The choice of dark adaptation duration and flash strength depends upon one of three aims (Fig. 1):

  1. To isolate the cone-mediated x-wave (peak time 30–50 ms): short dark-adaptation of around 5 min reveals the x-wave before it is masked by full development of the later rod-mediated b-wave [6, 8, 17].
  2. To separate the x- and b-wave peak times: if an ISCEV standard period of at least 20-min dark adaptation is used, weaker red flash strengths of around 0.03–0.3 cd s m^−2^ allow maximum separation in time of the cone- and rod-mediated components.
  3. To match the amplitudes of the DA red flash ERG b-wave with the ISCEV standard DA 0.01 ERG (rod ERG) b-wave, different red flash strengths may be needed depending upon the patient’s age, as the DA red flash rod b-wave diminishes with age, relative to the DA red flash x-wave [18].
- Frequency of red flash presentation. The inter-stimulus interval will influence the light adaptation of the retina and shape of the DA red flash ERG waveform [19]. The ISCEV standard for the DA 0.01 ERG is less than or equal to 1 flash every 2 s, and a similar frequency may be appropriate for flash strengths that elicit responses of similar amplitude to the DA 0.01 ERG.

## Calibration

Calibration is in accordance with the ISCEV ERG standard [1]. A spectral photometer is required to determine the spectral characteristics of the red flash. Stimulators may use different combinations of LEDs for different flash strengths, so equal spectral characteristics should not be assumed.

## Protocol specification

**Fig. 2:**
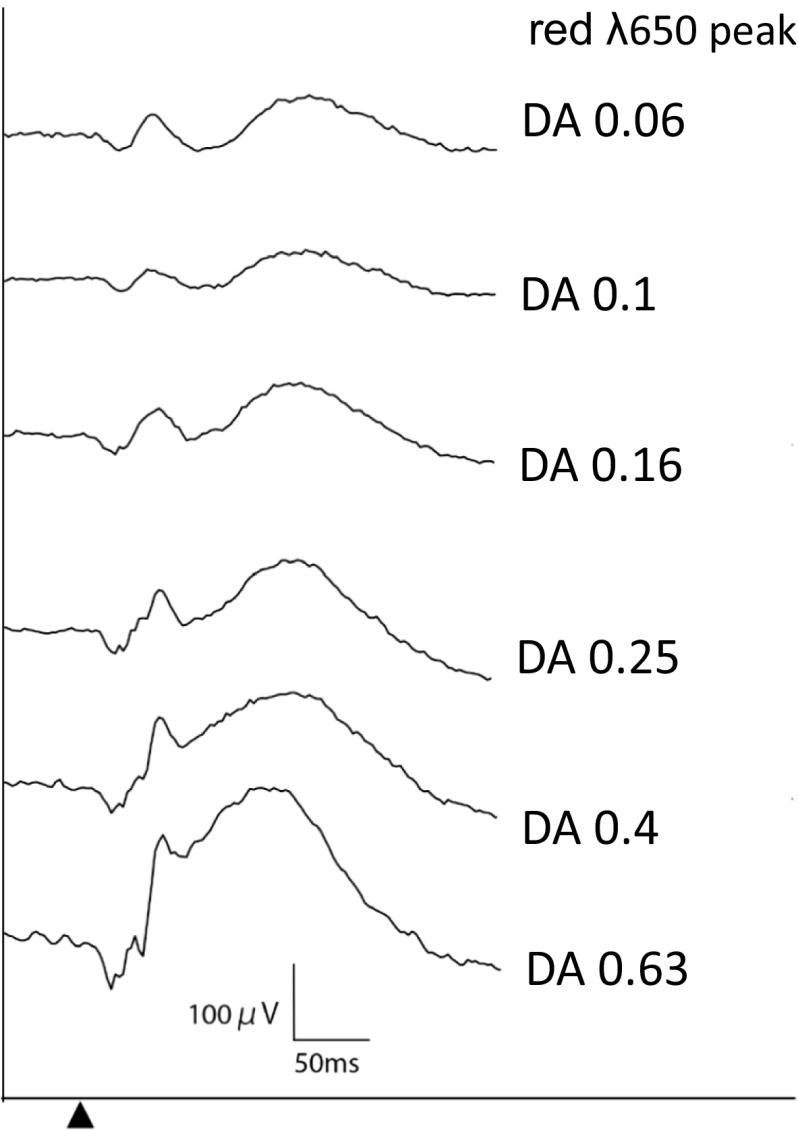
The gradual change in waveform of DA red flash ERG to a range of flash strengths weaker or stronger than 0.3 cd s m^−2^, recorded after 20 min DA with a 650 nm red flash
- *Flash strength* The minimum dark-adapted red flash protocol includes a red flash strength of 0.3 cd s m^−2^. This does not preclude the recording of additional red flash ERGs (ranging around 0.3 cd s m^−2^; see Fig. 2), but care should be taken to avoid light-adapting the retina with higher flash strengths, and it may be necessary to increase the inter-stimulus interval. If an additional red flash is defined as that required to elicit a DA red flash ERG rod system b-wave of equal or similar amplitude to the DA 0.01 ERG or to a DA blue flash ERG, this should be acknowledged and the corresponding flash stimuli stated in phot cd s m^−2^.
- *Duration of dark adaptation* The duration of dark adaptation required to record a dark-adapted red flash depends on the aims. The DA red flash ERG can be incorporated conveniently within the ISCEV standard ERG protocol after a minimum of 20-min DA, after the standard DA 0.01 ERG. Shorter DA times may be desirable to shorten the recording time or specifically to reduce the rod system b-wave and increase the prominence of the cone system x-wave (see above). Mesopic cone–rod interactions associated with shorter DA may increase the variability of the DA red ERG b-wave amplitude.
- *Frequency of red flash presentation* A flash rate of ≤ 0.5/s is recommended (inter-stimulus interval ≥ 2 s). This conforms to the current ISCEV standard for the DA 0.01 ERG. Longer inter-stimulus intervals may be needed for stronger red flashes.

Patient preparation follows that of the current ISCEV ERG standard [1] and the DA red flash ERG may be embedded within the standard ERG protocol. Additional specifications are listed below:

- *Stimulus wavelength* For routine diagnostic applications, LEDs with a peak wavelength of between 635 nm (Fig. 1) and 650 nm (Fig. 2) are suggested to allow separation of x- and b-waves. If Xenon flashes and filters are used, a dominant wavelength of 619 nm (e.g., Wratten 26) or 630 nm (e.g., Wratten 29) may be used. The peak wavelength and bandwidth at half-height of the stimulus and method of generation (optical filter or LED) should be stated.

## Response evaluation

Examples are shown in Figs. 1 and 2 of the DA red flash ERG waveforms produced by different flash strengths delivered using an LED (peak wavelength 635 or 650 nm). For routine testing, it is recommended that the x-wave and b-wave peak times and amplitudes are measured and reported. Peak times are measured from the flash (mid-point) and amplitudes from the baseline or a-wave (earliest negative trough) if present.

## Reporting

Reporting the DA red flash should follow the recommendations of the ISCEV ERG protocol. The flash stimulus characteristics (LED or filter), peak wavelength or filter specification (e.g., Wratten 26 or 29) should be stated. The flash strength should be stated. Unless already embedded within the ISCEV standard ERG protocol, pupil size and duration of dark adaption should be stated. The amplitude of the a-wave, x-wave and b-wave and their respective time to peaks may be reported along with age-appropriate laboratory reference data. It is acknowledged that in studies involving ISCEV standard ERGs it may be sufficiently informative to describe the relative reduction or preservation of x-wave and/or b-waves relative to each other and reference ranges.

## Appendix: Justification for the protocol details

We followed the Preferred Reporting Items for Systematic Reviews and Meta-Analyses (PRISMA) [20] when writing this report. The search strategy aimed to identify reports of scotopic red flash ERGs in order to extract stimulus parameters of wavelength, flash strength, stimulus duration, temporal frequency, dark adaptation period and amass evidence of its clinical application and range of response expected in normal and clinical cases.

A systematic literature review was performed to find publications that reported the scotopic red flash ERG initially from the period January 1942 to April 10, 2017 using MEDLINE, EMBASE and Cochrane reviews. Exclusion criteria were animal studies and absence of any stimulus specification. In summary, red flash strengths used, included 0.05, 0.1 [7], 0.17 [21], 0.25, 0.5, 0.75, 1 & 1.5 [22], 2.37 [23], 2.4 [2], 1.5 and 2.5 [24], and 8 cd s m^−2^ [25] (personal communication Sauve 2017) after 20 min DA. A range of Grass stroboscope flash settings have been stated as 1, 4, 8 and 16 e.g. gr4 white PS22 ~ 3.7 × 10^5^ candles [26] or gr4 + Wratten 26 filter = 0.02 Log μJ/cm^2^-steradian [18]. Some studies report flash strengths “such that in a normal subject the amplitude of the rod component to the red flash ERG is equivalent to that of the rod-specific response to a dim white flash (dark-adapted 0.01 cd s m^−2^)” [11, 12, 15, 27] or report that the red flash luminance empirically be set to elicit a b-wave of ~ 200μV [28]. The DA red flash ERG x-wave is reported to be stable between 10–70 years of age whilst the rod-driven b-wave diminishes with increasing age, [18]. To match b-wave amplitudes in the DA 0.01 and DA red flash ERGs it may be necessary to increase the red flash strength in older individuals. The main published parameters are summarized in Table 1.

**Table 1:** A summary of published DA red flash ERG parameters

Data	Peak *λ*	Flash strength	DA duration	LED/Xenon
Auerbach and Burian [6]	Wratten 29 635 nm Wratten 70 650 nm	6 and 12 cd s m^−2^	5 min	Xenon @30 cm
Francois et al. [9]	Neon 570 nm	0.1 J		Neon 0.2 s (orange)
	x-wave 25–60 μV@40 ms			
Iiyami and Yamaguchi [29]	Wratten 29 > 600 nm	86–112 cd s m^−2^	30 min	
Kellner and Foerster [10]	Wratten 29 623 nm	Not stated		Xenon in Ganzfeld
Lovasik et al. [23]	Wratten 26 > 600 nm	2.37 cd s m^−2^	Not stated	Xenon in Ganzfeld
*From figure*		90 μV@50 ms		
Mizunoya et al. [21]	LED 660 nm	0.17 cd s m^−2^	20 min	Contact lens Ganzfeld
Verdon et al. [19]	Wratten 26 > 600 nm			Xenon in Ganzfeld
*From figure*		@40 ms		
Lim and Ohn [2]	Wratten 26 = 605 nm (Scot match—14Db blue)	2.4 cd s m^−2^	45 min	Xenon in Ganzfeld
*Control data*		172.4 µV@46 ms		*N* = 52 adult
Weleber [18]	Wratten 26 > 600 nm	Gr1, 4 and 16	30 min	Xenon in Ganzfeld
*Control data*	Burian Allen	Gr1 = 50 µV (25–75) @40–50 ms Gr4 = 150 µV@50 ms Gr16 = 325 µV (200–400) @ 50 ms		*N* = 24 adult
Kriss et al. [8]	Grass red	Gr4	5 min	Grass @30 cm
	Peak 670 nm			
*Control data*	Skin electrode	14.3 (SD 4.9) µV@ 40.4 ms (SD 2.6) lower limit 4.5 µV @46.9		*N* = 30 over 5 m and adult
Chen et al. [22] abs	Espion	0.25 cd s m^−2^	20 min	LED
	color dome			
	Peak 635 nm			
*Control data*	a-wave	17.6 µV@19.8 ms		*N* = 37 adult
	x-wave	64 µV@50.7 ms		
	b-wave	68 µV@72.9 ms		
Hamilton and Graham [17]	Skin electrode	0.2–2.0 cd s m^−2^	20 min	*N* = 16 adults
